# Supplementary material for: Phase-engineered cathode for super-stable potassium storage
Source: Nat Commun. 2023 Feb 6;14:644. doi: 10.1038/s41467-023-36385-4 (PMC9902589; doi:10.1038/s41467-023-36385-4)
Supplement: Supplementary file 1 — Supplementary Information [file 41467_2023_36385_MOESM1_ESM.pdf]

Supplementary information for

Phase-engineered cathode for super-stable potassium storage

Lichen Wu[1,2], Hongwei Fu[1,2], Shu Li[1,2], Jian Zhu[3]*, Jiang Zhou[4], Apparao M. Rao[5], Limei Cha[6,7,8]*, Kunkun Guo[9], Shuangchun Wen[1], Bingan Lu[1,2]*

[1] School of Physics and Electronics, Hunan University, Changsha 410082, P. R. China.

[2] State Key Laboratory of Advanced Design and Manufacturing for Vehicle Body, Hunan University, Changsha 410082, P. R. China.

[3] College of Chemistry and Chemical Engineering, Hunan University, Changsha 410082, P. R. China.

[4] School of Materials Science and Engineering, Central South University, Changsha 410083, P. R. China.

[5] Department of Physics and Astronomy, Clemson Nanomaterials Institute, Clemson University, SC 29634, USA.

[6] Materials Science and Engineering program, Guangdong Technion–Israel Institute of Technology, Shantou 515063, P. R. China.

[7] Materials Science and Engineering program, Technion–Israel Institute of Technology, Haifa 32000, Israel.

[8] MATEC key lab, Guangdong Technion–Israel Institute of Technology, Shantou 515063, P. R. China.

[9] College of Materials Science and Engineering, Hunan University, Changsha 410082, P. R. China.

*Corresponding author. E-mail: jzhu@hnu.edu.cn(J.Z.); cha.limei@gtiit.edu.cn

(L.C.); luba2012@hnu.edu.cn (B.L.)

This file includes:

Supplementary Figures 1 to 23

Supplementary Tables 1 to 3

Supplementary References

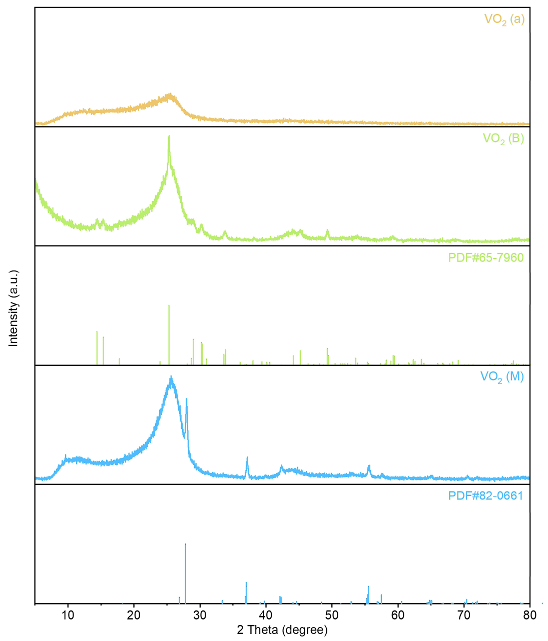

Supplementary Figure 1. XRD patterns and the corresponding JCPDS data of VO_2 (a), VO_2 (B), and VO_2 (M).

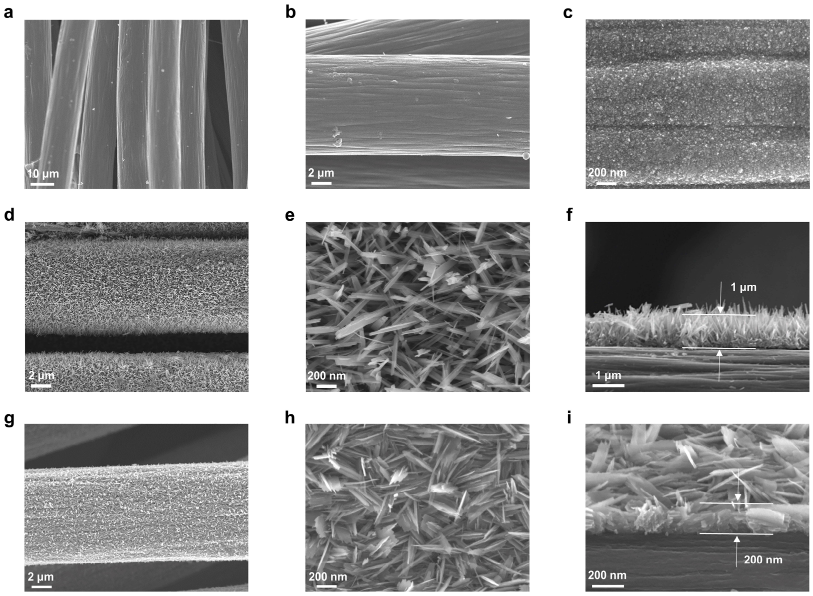

Supplementary Figure 2. FE-SEM images for the several types of VO_2 on the carbon fiber cloth. Low-magnification FE-SEM images of a), b) VO_2 (a), d) VO_2 (B), and g) VO_2 (M). High-magnification FE-SEM images of c) VO_2 (a), e) VO_2 (B), and h) VO_2 (M). Cross-sectional FE-SEM images of f) VO_2 (B) and i) VO_2 (M).

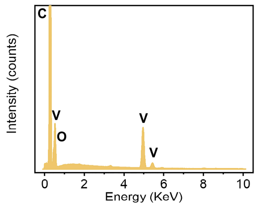

Supplementary Figure 3. EDS results of VO_2 (a).

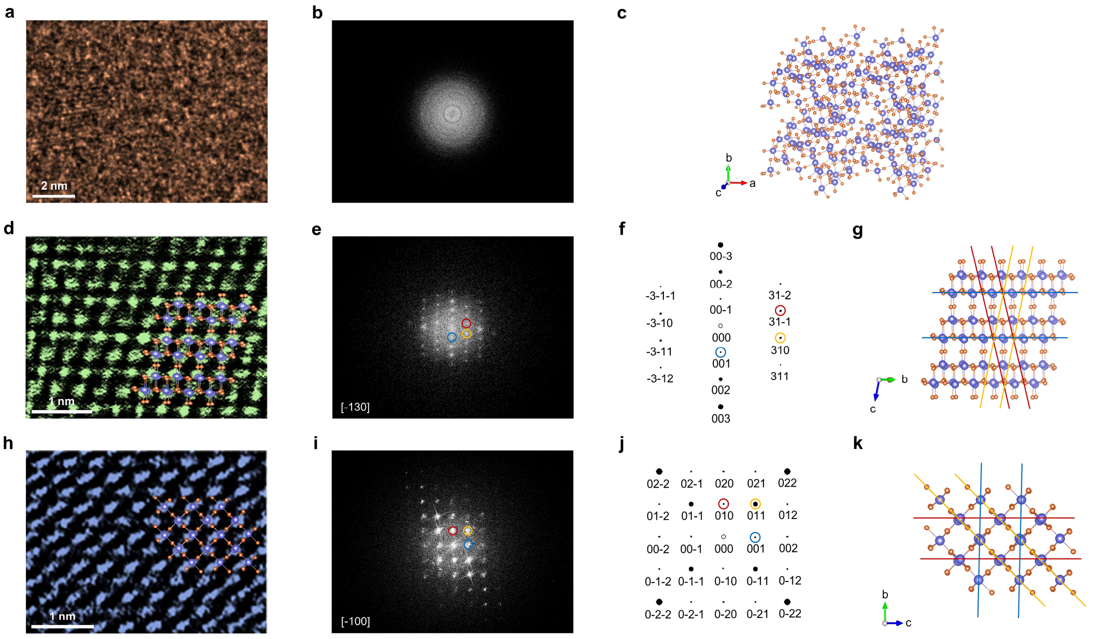

Supplementary Figure 4. Morphology and structural characterization of the pristine materials. The HRTEM images at atomic resolution of pristine a) VO_2 (a), d) VO_2 (B), and h) VO_2 (M). Their corresponding FFT patterns are shown in b), e), and i), respectively. Schematic illustrations of c) VO_2 (a), g) VO_2 (B), and k) VO_2 (M). The simulated diffraction patterns of f) VO_2 (B) and j) VO_2 (M).

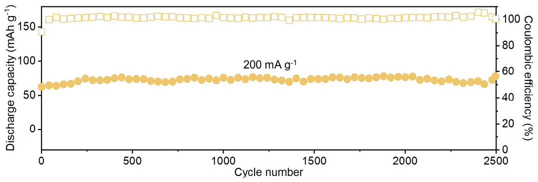

Supplementary Figure 5. Cycle life performance of VO_2 (a) at 200 mA g^{-1}.

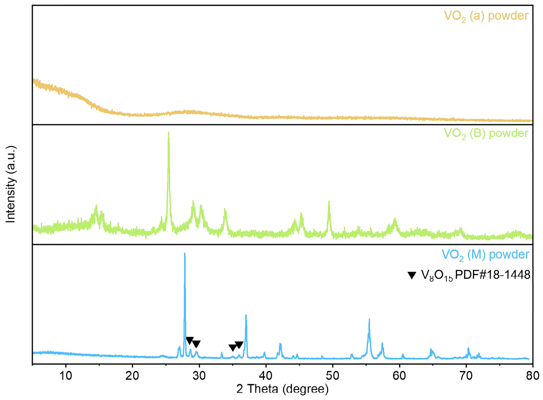

Supplementary Figure 6. XRD patterns of VO_2 (a) powder, VO_2 (B) powder, and VO_2 (M) powder.

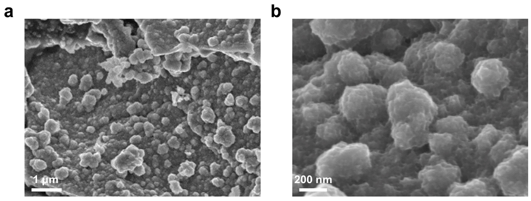

Supplementary Figure 7. Material characterizations of amorphous VO_2 powders.

a) and b) The FE-SEM images of amorphous VO_2 powder.

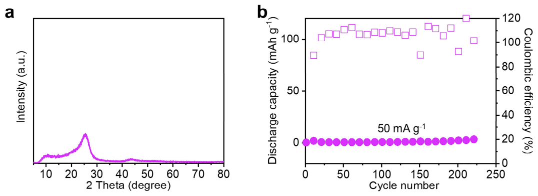

Supplementary Figure 8. Material characterization and electrochemical performance of pure CF. a) XRD pattern and b) cycle performance at 50 mA g^{-1} for pure CF.

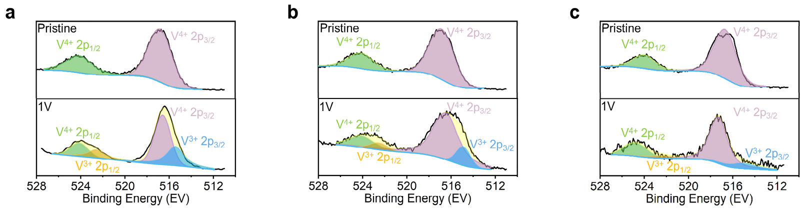

Supplementary Figure 9. XPS spectra of V 2*p* of a) VO_2 (a), b) VO_2 (B) and c) VO_2 (M) at pristine state and discharged state of 1 V.

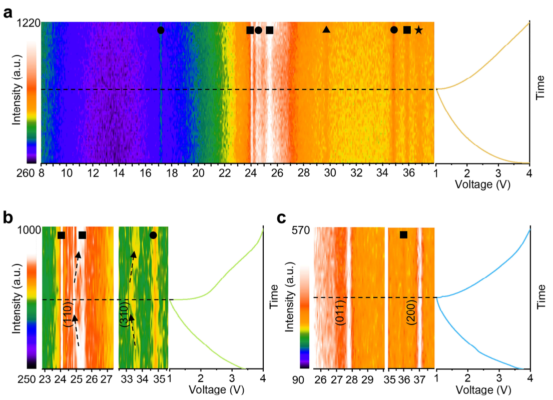

Supplementary Figure 10. In situ XRD patterns of a) VO_2 (a), b) VO_2 (B), and c) VO_2 (M) half cells during a cycle and the corresponding galvanostatic curves. The peak positions marked by the following symbols are due to the Be disk: ▲: $Be_{11}Fe$; ●: $Be_{12}Cr$; ■: Be_2Cr; ★: BeS.

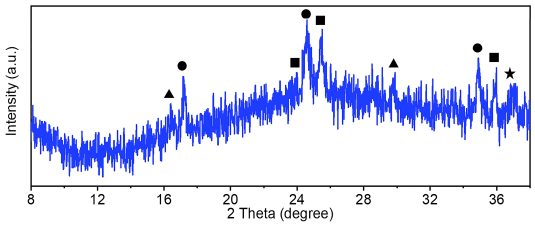

Supplementary Figure 11. *In situ* XRD test with a blank sample. The peaks labeled with different symbols on the peaks correspond to: ▲: $Be_{11}Fe$; ●: $Be_{12}Cr$; ■: Be_2Cr; ★: BeS.

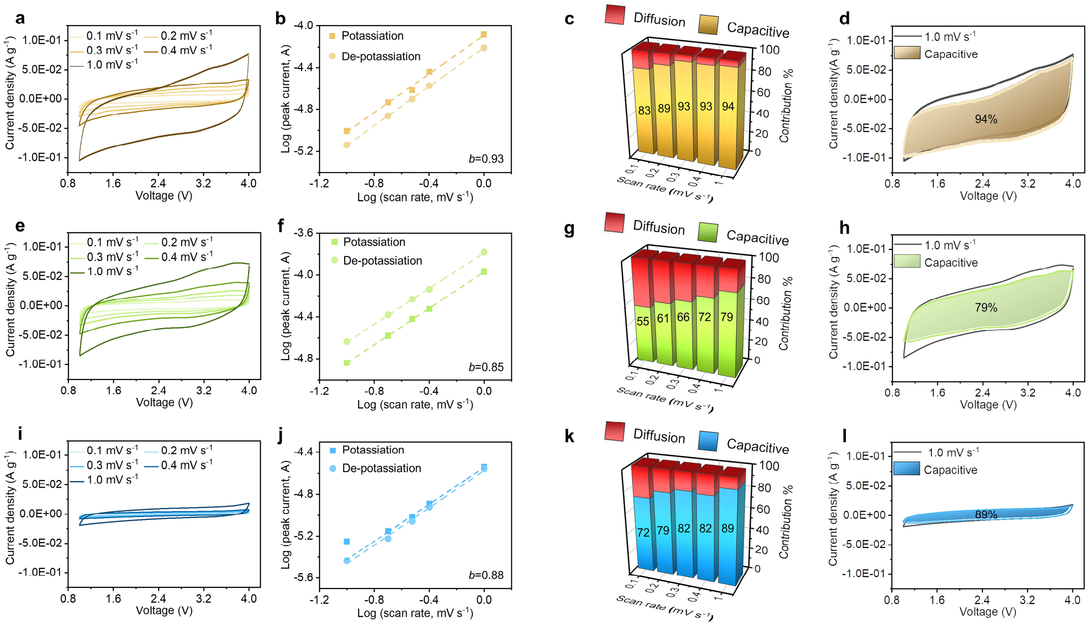

Supplementary Figure 12. The electrokinetic analyses conducted by CV test. a), e), and i) CV curves for VO_2 (a), VO_2 (B), and VO_2 (M) at different scan rates. b), f) and j) Relationship between the logarithmic scan rates and logarithmic peak currents. c), g) and k) Contribution of pseudocapacitive capacities for K^+ storage at different scan rates. d), h), and l) The capacitive fractions at a scan rate of 1.0 mV s^{-1} for VO_2 (a), VO_2 (B), and VO_2 (M), respectively.

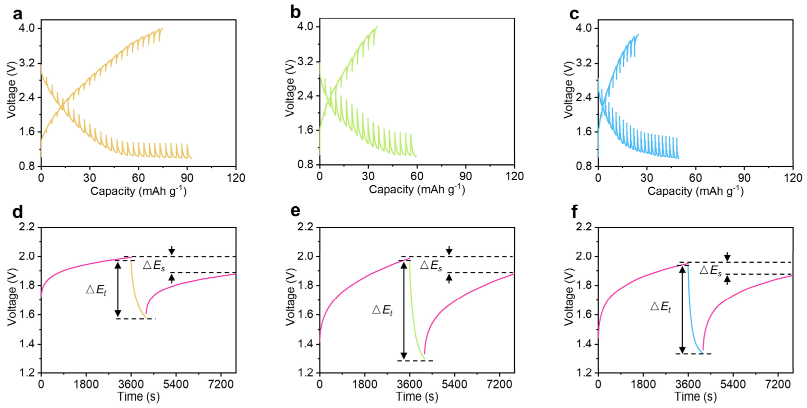

Supplementary Figure 13. Investigation of the K^+ ions migration kinetics by GITT. Transient voltage profiles versus specific capacity for potassiation/de-potassiation in a) VO_2 (a), b) VO_2 (B), and c) VO_2 (M) obtained from GITT. A single step of GITT for d) VO_2 (a), e) VO_2 (B), and f) VO_2 (M) during the initial discharge process.

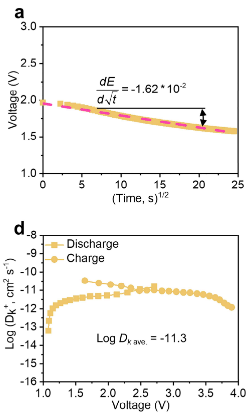

Supplementary Figure 14. The chemical diffusion coefficient D_K calculated by GITT. The representation transient voltage of a galvanostatic pulse as a function of the square root of time from GITT for a) VO_2 (a), b) VO_2 (B), and c) VO_2 (M). The calculated chemical diffusion coefficient D_K versus voltage for d) VO_2 (a), e) VO_2 (B), and f) VO_2 (M), respectively.

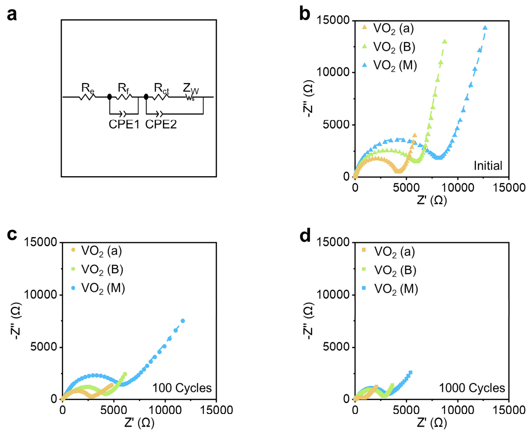

Supplementary Figure 15. The electrochemical impedance spectroscopy. a) An equivalent circuit model for the impedance spectra. Nyquist plots for VO_2 in different phases at b) open-circuit voltage states, c) charged states after 100 cycles, and d) charged states after 1000 cycles.

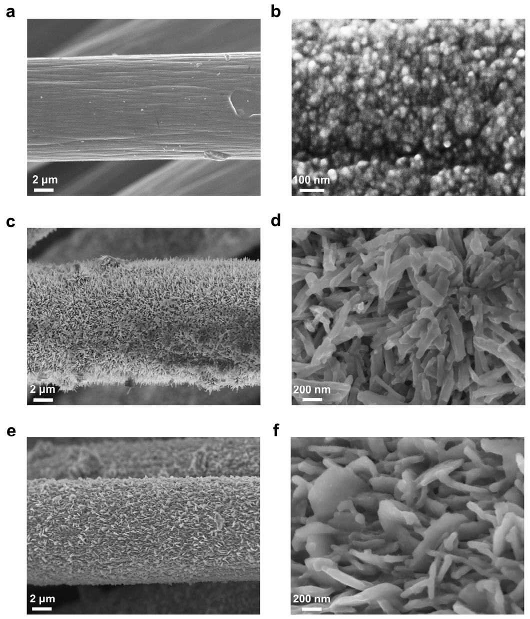

Supplementary Figure 16. The FE-SEM images after cycling. FE-SEM of a), b) VO_2 (a), c), d) VO_2 (B), and e), f) VO_2 (M) after 100 cycles at charged states.

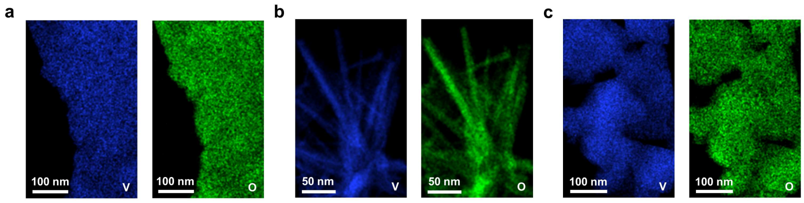

Supplementary Figure 17. Elemental mappings of V and O for a) VO_2 (a), b) VO_2 (B), and c) VO_2 (M).

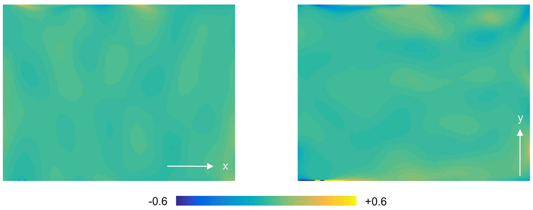

Supplementary Figure 18. Strain mapping with uniaxial strain components ε_{xx} (left) and ε_{yy} (right), as obtained by GPA of the HRTEM image for pristine VO_2 (B).

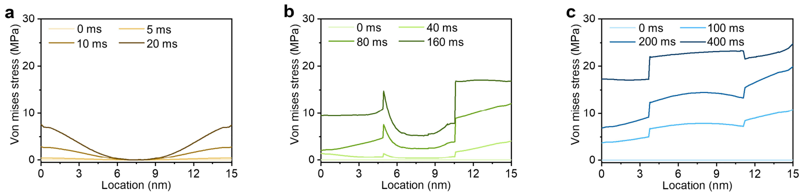

Supplementary Figure 19. Von Mises stress within the a) VO_2 (a), b) VO_2 (B), and c) VO_2 (M) particles at different times along the radial direction.

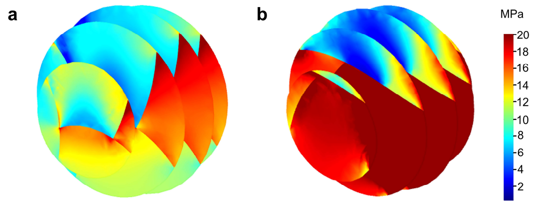

Supplementary Figure 20. 3D view of equivalent stress after K^+ ions diffusion in a) VO_2 (B) particle for 160 ms, and b) VO_2 (M) particle for 400 ms, respectively.

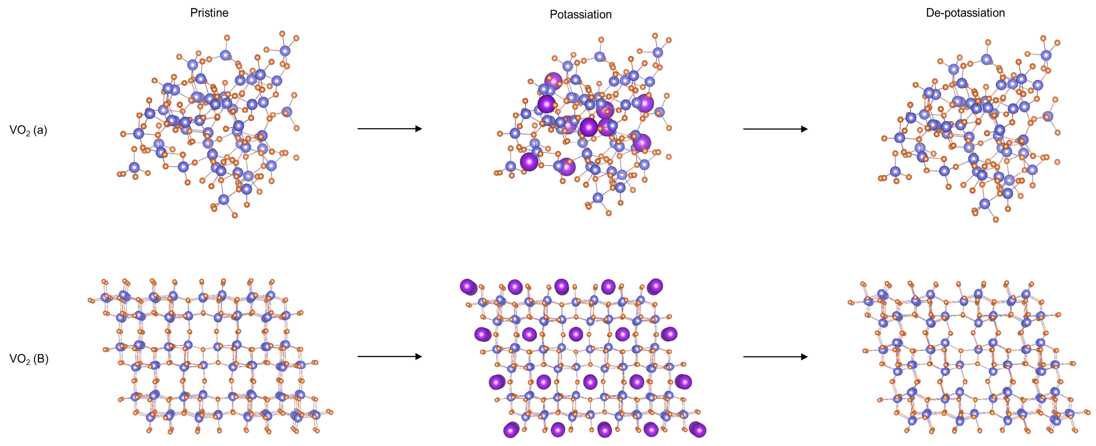

Supplementary Figure 21. The schematic structural changes for VO_2 (a) and VO_2 (B) during the potassiation and depotassiation.

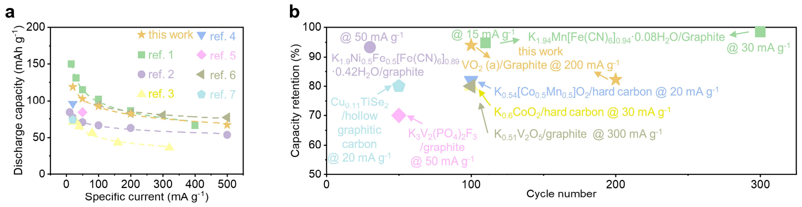

Supplementary Figure 22. Comparison of full battery a) rate capability and b) cycling performance of VO_2 (a) as the cathode with the reported systems[1-7].

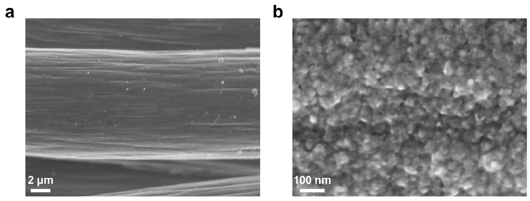

Supplementary Figure 23. The FE-SEM images of VO_2 (a) after bending 1000 times at charged states.

Supplementary Table 1. The atomic ratio of elements from EDS.

Element	Wt %	Atomic %
C	76.09	87.92
O	9.37	8.12
V	14.54	3.96

Supplementary Table 2. The fitting results of EIS for VO_2 in different phases.

Sample	Item	Initial	100 cycles	1000 cycles
VO_2 (a)	R_e (Ω)	6.319	15.74	22.93
	R_f (Ω)	1.45	127.5	93
	R_{ct} (Ω)	3904	2414	756.7
VO_2 (B)	R_e (Ω)	6.08	7.123	8.144
	R_f (Ω)	2.407	754.8	470.4
	R_{ct} (Ω)	5421	3462	2315
VO_2 (M)	R_e (Ω)	8.008	10.13	9.684
	R_f (Ω)	1.258	1245	483.8
	R_{ct} (Ω)	8345	4418	2534

Supplementary Table 3. The density test results for amorphous VO_2 powders.

Sample	Volume (cm^3)	Density ($g\ cm^{-3}$)	Elapsed Time (mm:ss)	Temperature (°C)
1	0.1802	2.8075	11:13	31.14
2	0.1800	2.8100	13:46	31.18
3	0.1797	2.8141	16:15	31.24
4	0.1804	2.8031	18:49	31.29
5	0.1809	2.7961	21:22	31.29

Supplementary References

1 Deng, L. *et al.* Defect-free potassium manganese hexacyanoferrate cathode material for high-performance potassium-ion batteries. *Nat. Commun.* **12,** 2167 (2021).

2 Chong, S. *et al.* Potassium nickel iron hexacyanoferrate as ultra-long-life cathode material for potassium-ion batteries with high energy density. *ACS Nano* **14,** 9807-9818 (2020).

3 Deng, T. *et al.* Self-templated formation of P2-type $K_{0.6}CoO_2$ microspheres for high reversible potassium-ion batteries. *Nano Lett.* **18,** 1522-1529 (2018).

4 Choi, J. U. *et al.* $K_{0.54}[Co_{0.5}Mn_{0.5}]O_2$: New cathode with high power capability for potassium-ion batteries. *Nano Energy* **61,** 284-294 (2019).

5 Lin, X., Huang, J., Tan, H., Huang, J. & Zhang, B. $K_3V_2(PO4)_2F_3$ as a robust cathode for potassium-ion batteries. *Energy Storage Mater.* **16,** 97-101 (2019).

6 Zhu, Y.-H. *et al.* Reconstructed orthorhombic V_2O_5 polyhedra for fast ion diffusion in K-ion batteries. *Chem* **5,** 168-179 (2019).

7 Wu, L. et al. Layered superconductor $Cu_{0.11}TiSe_2$ as a high-stable K-cathode. *Adv. Funct. Mater.*, 2109893 (2021).
